# Supplementary material for: Polyketide synthase gene clusters in the ericoid mycorrhizal fungus Oidiodendron maius: genome-wide prediction, expression profiling, and functional characterization of knockout mutants
Source: Front Plant Sci. 2026 Sep 16;17:1924430. doi: 10.3389/fpls.2026.1924430 (PMC13623903; doi:10.3389/fpls.2026.1924430)
Supplement: Supplementary file 1 [file SupplementaryFile1.zip › Supplementary Materials/Supplementary Folder 1.zip!/Oidma1_AssemblyScaffolds_Repeatmasked/knownclusterblast/cluster55/jgi.p_Oidma1_138794_mibig_hits.html]

| MIBiG Protein | Description | MIBiG Cluster | MiBiG Product | % ID | % Coverage | BLAST Score | E-value |
| --- | --- | --- | --- | --- | --- | --- | --- |
| EAL89337.1 | O-methyltransferase,\_putative | BGC0001403 | Trypacidin\_biosynthetic\_gene\_cluster | 71 | 99.7933884298 | 694 | 1e-199 |
| CCE67071.1 | methyltransferase | BGC0001242 | Fusarubin\_biosynthetic\_gene\_cluster | 26 | 86.1570247934 | 114 | 3.4e-25 |
| ABK64180.1 | O-methyltransferase | BGC0001247\_c3 | Cercosporin\_biosynthetic\_gene\_cluster | 26 | 91.5289256198 | 113 | 7.5e-25 |
| BAE71329.1 | o-methyltransferase\_A | BGC0000004 | Aflatoxin\_biosynthetic\_gene\_cluster | 25 | 87.1900826446 | 99 | 8.6e-21 |
| AAM70356.1 | CalO6 | BGC0000033 | Calicheamicin\_biosynthetic\_gene\_cluster | 24 | 83.0578512397 | 96 | 7.3e-20 |
| AAD32742.2 | MmcR | BGC0000915 | Mitomycin\_biosynthetic\_gene\_cluster | 24 | 77.6859504132 | 96 | 9.5e-20 |
| AAS90087.1 | OmtA | BGC0000010 | Aflatoxin\_biosynthetic\_gene\_cluster | 25 | 84.2975206612 | 96 | 9.5e-20 |
| AAS90041.1 | OmtA | BGC0000008 | Aflatoxin\_biosynthetic\_gene\_cluster | 26 | 84.2975206612 | 96 | 1.2e-19 |
| AAS90104.1 | OmtA | BGC0000006 | Aflatoxin\_biosynthetic\_gene\_cluster | 25 | 84.5041322314 | 94 | 4.7e-19 |
| AAS90065.1 | OmtA | BGC0000009 | Aflatoxin\_biosynthetic\_gene\_cluster | 24 | 81.6115702479 | 90 | 5.2e-18 |
| EAL92292.1 | O-methyltransferase\_GliM-like,\_putative | BGC0000372 | Hexadehydro-astechrome\_(HAS)\_biosynthetic\_gene\_cluster | 30 | 41.3223140496 | 89 | 8.9e-18 |
| ACN64847.1 | PokMT3 | BGC0001061 | Polyketomycin\_biosynthetic\_gene\_cluster | 28 | 52.6859504132 | 86 | 9.8e-17 |
| AAS90018.1 | OmtA | BGC0000007 | Aflatoxin\_biosynthetic\_gene\_cluster | 31 | 38.8429752066 | 84 | 2.9e-16 |
| AEA29649.1 | putative\_O-methyltransferase | BGC0000409 | Porothramycin\_biosynthetic\_gene\_cluster | 31 | 45.041322314 | 84 | 3.7e-16 |
| AKD43502.1 | O-Methyltransferase | BGC0001409 | Dutomycin\_biosynthetic\_gene\_cluster | 28 | 53.305785124 | 83 | 6.4e-16 |
| AFD30960.1 | CrmM | BGC0000966 | Caerulomycin\_A\_biosynthetic\_gene\_cluster | 27 | 38.6363636364 | 71 | 2.5e-12 |
| AEE65480.1 | putative\_O-methyltransferase | BGC0000223 | Fluostatin\_biosynthetic\_gene\_cluster | 29 | 38.2231404959 | 67 | 3.6e-11 |
| ABV56592.1 | KtzL | BGC0000378 | Kutznerides\_biosynthetic\_gene\_cluster | 34 | 22.3140495868 | 62 | 1.5e-09 |
| AIT42115.1 | putative\_methyltransferase | BGC0001221 | Marinophenazines\_biosynthetic\_gene\_cluster | 29 | 21.694214876 | 51 | 3.5e-06 |
